# Influence of Developmental Conditions on Immune Function and Dispersal-Related Traits in the Glanville Fritillary (*Melitaea cinxia*) Butterfly

**DOI:** 10.1371/journal.pone.0081289

**Published:** 2013-11-22

**Authors:** Marjo Saastamoinen, Markus J. Rantala

**Affiliations:** 1 Department of Biological Sciences, University of Helsinki, Helsinki, Finland; 2 Department of Biology, University of Turku, Turku, Finland; USDA-Agricultural Research Service, United States of America

## Abstract

Organisms in the wild are constantly faced with a wide range of environmental variability, such as fluctuation in food availability. Poor nutritional conditions influence life-histories via individual resource allocation patterns, and trade-offs between competing traits. In this study, we assessed the influence of food restriction during development on the energetically expensive traits flight metabolic rate (proxy of dispersal ability), encapsulation rate (proxy of immune defence), and lifespan using the Glanville fritillary butterfly, *Melitaea cinxia*, as a model organism. Additionally, we examined the direct costs of flight on individual immune function, and whether those costs increase under restricted environmental conditions. We found that nutritional restriction during development enhanced adult encapsulations rate, but reduced both resting and flight metabolic rates. However, at the individual level metabolic rates were not associated with encapsulation rate. Interestingly, individuals that were forced to fly prior to the immune assays had higher encapsulation rates than individuals that had not flown, suggesting that flying itself enhances immune response. Finally, in the control group encapsulation rate correlated positively with lifespan, whereas in the nutritional restriction group there was no relationship between these traits, suggesting that the association between encapsulation rate on adult lifespan was condition-dependent. Thus stressful events during both larval development (food limitation) and adulthood (forced flight) induce increased immune response in the adult butterflies, which may allow individuals to cope with stressful events later on in life.

## Introduction

Parasites and pathogens are pervasive and cause substantial fitness costs to their hosts. Therefore hosts have evolved effective immune systems. However, both the maintenance and the activation of an immune system are energetically expensive [1–3] and therefore trade-offs between immune defence and other life-history traits are expected [4]. The extent to which an organism should invest in its immune defence depends on the efficiency of the defence, the risk of being attacked and the magnitude of costs associated with mounting an immune response [5]. Individual’s nutritional resources and body condition can influence immune investment directly, for example by individuals with fewer resources being able to allocate less to immunity [6–8]. Subsequent strategic decisions related to other life-history traits [9] can further influence immunity, for example in the form of increased trade-offs between immunity and other expensive life history traits [2]. 

One key life-history trait in many organisms is dispersal, as it determines the potential spread of individuals and populations, and by means of gene flow it can also influence the rate of adaptation to changing conditions [10]. Similarly to immune defence, dispersal is an energetically expensive trait [11]. Studies testing the relationship between dispersal or flight and immune defence are relatively scarce. However, it was recently shown that in a damselfly, *Calopteryx splendens*, an activation of the immune system increases dispersal rate, suggesting that immune function may play an important role in the evolution of dispersal [12]. On the contrary, in bumblebees (*Bombus terrestris*; foraging activity) and in crickets (*Gryllus texensis*; tethered flight) energetic activities have been shown to reduce immune defence, indicating a life history trade-off between these two traits [13,14]. 

One possible mechanism for flight to impact immune defence is via its effect on metabolism. In the Glanville fritillary butterfly, for example, one-third of the variation in the distance moved in one hour is attributable to variation in flight metabolic rate [15]. However, the impacts of metabolic rate on immune defence are not, in general, consistent. For example, in a study of crickets [16] no correlation between metabolic rate and immune defence was found, whereas across seven *Drosophila* species immune defence was shown to be negatively associated with mass specific metabolic rate [17]. In the cabbage butterfly, individuals challenged with a nylon implant (mimicking parasitism) raised their standard metabolic rate nearly 8% compared to the control individuals [1]. 

Environmental conditions can have a great impact on individual performance. Food limitation during developmentally critical periods, for example, has been shown to have long lasting negative influences on numerous adult life history traits [9,18]. In the Glanville fritillary butterfly, we have previously shown that even short term food limitation during development can reduce adult lifespan and have other negative fitness consequences [19]. Similarly, the immune defence has been shown to be influenced by genetic [20] as well as environmental factors, such as nutrition [6,21]. Less is known about the condition-dependency of dispersal or flight metabolism, even though the importance of environmental factors in determining dispersal propensity is becoming increasingly recognised [6,22–24]. Finally, based on life history theory, trade-offs between energetically expensive traits may only be apparent under suboptimal environmental conditions [2,25].

Insects in general are excellent model organism for immuno-ecological studies because their immune defence system is far less complex than the vertebrate immune system, even though many components are homologous [26]. Insects defend themselves against foreign invaders such as parasites and pathogens by cellular encapsulation, antimicrobial peptides, prophenoloxidase cascade, and phagocytosis nodulation [27,28]. 

In the present study, our aim was to investigate the influence of short-term food limitation during development on four energetically expensive traits: encapsulation rate (immune defence), flight metabolic rate (dispersal ability), resting metabolic rate, and lifespan (proxy of adult fitness) using the Glanville fritillary (*Melitaea cinxia*) butterfly as a model organism. In addition, we examined the direct costs of induced immune response and flight on adult lifespan and whether those costs are greater under restrictive (i.e. food-limited) environmental conditions. 

## Materials & Methods

### Study species

In Finland, the Glanville fritillary (*Melitaea cinxia*) butterfly occurs only in the south-western archipelago, the Åland Islands. In the Åland Islands, the larvae feed on two host plant species, *Plantago lanceolata* and *Veronica spicata* [29], which occur in naturally fragmented dry meadows. The Glanville fritillary butterfly has a classical metapopulation structure in the Åland Islands, with a high rate of population turnover (extinctions and re-colonisations; [30]). Population persistence is therefore highly dependent on dispersal, as hundreds of new populations are established each year on habitat patches that were unoccupied in the previous year. These new populations compensate for the loss of a similar number of local populations due to extinction, making the metapopulation as a whole stable over time [30,31]. 

### Experimental set-up

For the experiment 399 larvae from 24 families were reared under common garden conditions in the laboratory (27:10°C; 12:12, L/D). These families were the F2 generation of butterflies collected from independent local populations in the Åland Islands. The butterfly is not classified as endangered or protected and hence no permits are required for the collection in the Åland Islands. The larvae were fed leaves of *Plantago lanceolata*. All individuals were weighed (Mettler-Toledo XS 105 analytical balance, accuracy 0.01 mg) at the beginning of the 7^th^ instar, after which the larvae were individually reared. At this stage larvae were randomly assigned to one of two nutritional treatments: control (food *ad libitum*; N = 206 larvae) and nutritional restriction (N = 193 larvae). In the “nutritional restriction” treatment the larvae experienced a total of three full days without food (0), with food (1) being provided in between the restricted days (the pattern followed was 10101011111). All individuals were weighed again one day after pupation. Three days after pupation, we assessed the immunity of 152 individuals (N = 81 and 71 for control and nutritional restriction, respectively). These individuals represented all families. After eclosion, the rest of the butterflies were sexed and individually marked by writing a number on the underside of the hind wing. Three days after eclosion, resting and flight metabolic rates of 156 butterflies were measured (control: 37 and 38 females and males, respectively; nutritional restriction: 38 and 41 females and males, respectively). Immediately after the measurement of the metabolic rates adult immunity was assessed. We also assessed adult immunity for 72 three-day old individuals that were not assessed for metabolic rates (control: 19 and 21 females and males, respectively; nutritional restriction: 15 and 17 females and males, respectively). After the immunity assay, the adult butterflies were kept in cylindrical cages (diameter = 40 cm, height = 50 cm) under standard conditions (27:10°C; 12:12, L/D) to assess their lifespan. Individuals were fed daily with honey:water solution (1:4) until they died. On the day of the flight metabolic rate and/or immunity assessment individuals were given food only after the assay.

#### Pupal immune response

As a measure of immune response we assessed encapsulation, which is a non-specific, constitutive, cellular response through which insects defend themselves against multicellular pathogens such as nematodes, fungi and parasitoids [27]. It also plays a role in defence against viruses [32] and some bacteria [33]. In the Glanville fritillary butterfly, encapsulation is known to work at least against a specialist parasitoid [34] and there is also a significant correlation between encapsulation rate and survival to bacterial infection [35]. Encapsulation rate was measured by inserting a 2 ± 0.1 mm long piece of nylon monofilament (diameter 0.18 mm, rubbed with sandpaper) through a puncture in the pupal cuticle [20]. A knot on the monofilament ensured that an equally long filament was inserted into each individual. After insertion of the monofilament, pupae were placed in individual Eppendorf tubes and kept at a constant temperature (+27 ± 1 °C) for 1 h to allow for an immune response. Our preliminary experiments indicated that 1 h gave the highest variance between individuals in encapsulation rate, while still not at an equilibrium (data not shown). At the end of this period pupae were frozen at -80 °C. For the encapsulation analyses, the monofilament implant was removed and photographed from three different angles under a light microscope. These pictures were analysed using the ImageJ program (National Institutes of Health, USA). The degree of encapsulation was analysed as grey values of reflecting light from the implants. For each sample we used the average grey values of three pictures for the analyses. The data were transformed so that the darkest grey values correspond to the highest encapsulation rate. This transformation was done by subtracting the observed grey values from the control grey value (clear implant; see 36,37 for details). 

#### Metabolic rates

Flight metabolic rate was measured using standard respirometry techniques [38]. Butterflies were stimulated to fly inside a transparent 1 L (diameter 12 cm) jar through which dry CO_2_-free air was pumped at a regulated flow rate of 1.0 L min^-1^. A thermal sensor inside the jar recorded air temperature at a 1Hz sampling frequency (Sable Systems UI2 interface). Air temperature within the jar was nearly invariant during the testing of an individual butterfly, averaging +30.2 °C across all tests. Gentle shakes or taps were applied to get the butterflies flying again whenever they alighted. Flight was stimulated for 10 min, after which the jar was shaded and a steady baseline of resting CO_2_ emission was re-established. In the cases were individuals stopped flight prior to the end of the 10 min assay, we continued stimulation so that the butterfly flew again as soon as it was able to. At the end of the assay the butterfly was removed from the jar and its adult immune response was assessed. The respirometry experiments were performed blindly with regard to the treatment of the butterflies. From the recorded data, the mean pre-flight CO_2_ emission rate (Resting metabolic rate) was subtracted to determine the rate of CO_2_ emission attributable to flight metabolism. We assessed both peak and total CO_2_ production during the flight, as these two variables may measure different functions of dispersal. 

#### Adult immune response

To measure encapsulation rate, adults were first chilled for 5 minutes at + 5 C°. They were then placed with open wings on soft foam plastic. A piece of mesh was pinned on top of the butterfly to prevent it from moving. We then inserted a 2 ± 0.1 mm long piece of nylon monofilament (diameter 0.18 mm, rubbed with sandpaper) through a puncture in the centre of the thorax cuticle. After insertion of the monofilament adults were kept motionless at constant temperature (+27 ± 1 °C) for 1 h to allow for an immune response. Our preliminary experiments indicated that, as for the pupae, 1 h gave the highest variance between individuals in encapsulation rate, while still not at an equilibrium (data not presented). At the end of this period the filament was removed by sliding it through the puncture, and stored at -80 °C. After removal of the filament individuals were allowed to imbibe 25% honey solution, and were returned to the cage. Preliminary experiments had shown that assessment of the encapsulation rate in adults does not affect their lifespan (data not shown). Encapsulation rate was analysed as explained above for pupal immunity assay.

### Data analyses

Linear mixed model approaches (SAS v. 9.2. for Windows; SAS Institute, Cary, NC, USA) were used to examine the influence of larval food treatment on individual development, encapsulation rate (pupal and adult stages), metabolic rates and adult lifespan. The developmental traits included length of the final instar, increase in mass during the final instar, pupal mass and length of pupal stage. The metabolic rates included resting metabolic rate and peak and total metabolic rate during flight. Mass-corrected measures of metabolic rates were obtained by regressing the metabolic rate measures against pupal mass and using the residuals as explanatory variables. Most of the data were normally distributed. The length of pupal stage was analysed with binomial distribution and logit link function, resting metabolic rate was log transformed to reach normality, and adult encapsulation rate was analysed with gamma distribution and log link function. 

The explanatory variables were treatment and sex, except in the analyses of pupal encapsulation rate, as pupae were killed after the assays so we were unable to determine their sex. For pupal encapsulation rate we included pupal mass and increase in body mass during the final instar as covariates. For the metabolic rate analyses temperature during the highest CO_2_ peak was included as a covariate. For the adult encapsulation and lifespan we also assessed the influence of metabolic rates and whether or not individuals experienced the forced flight treatment. Finally, for the lifespan analysis we tested the influence of adult encapsulation rate. Family was included as a random factor in all analyses. Initial models included all second order interactions. They were then simplified by the removal of non-significant interaction terms to give a final minimal adequate model. 

## Results

### Development time and pupal mass

**Figure 1:**
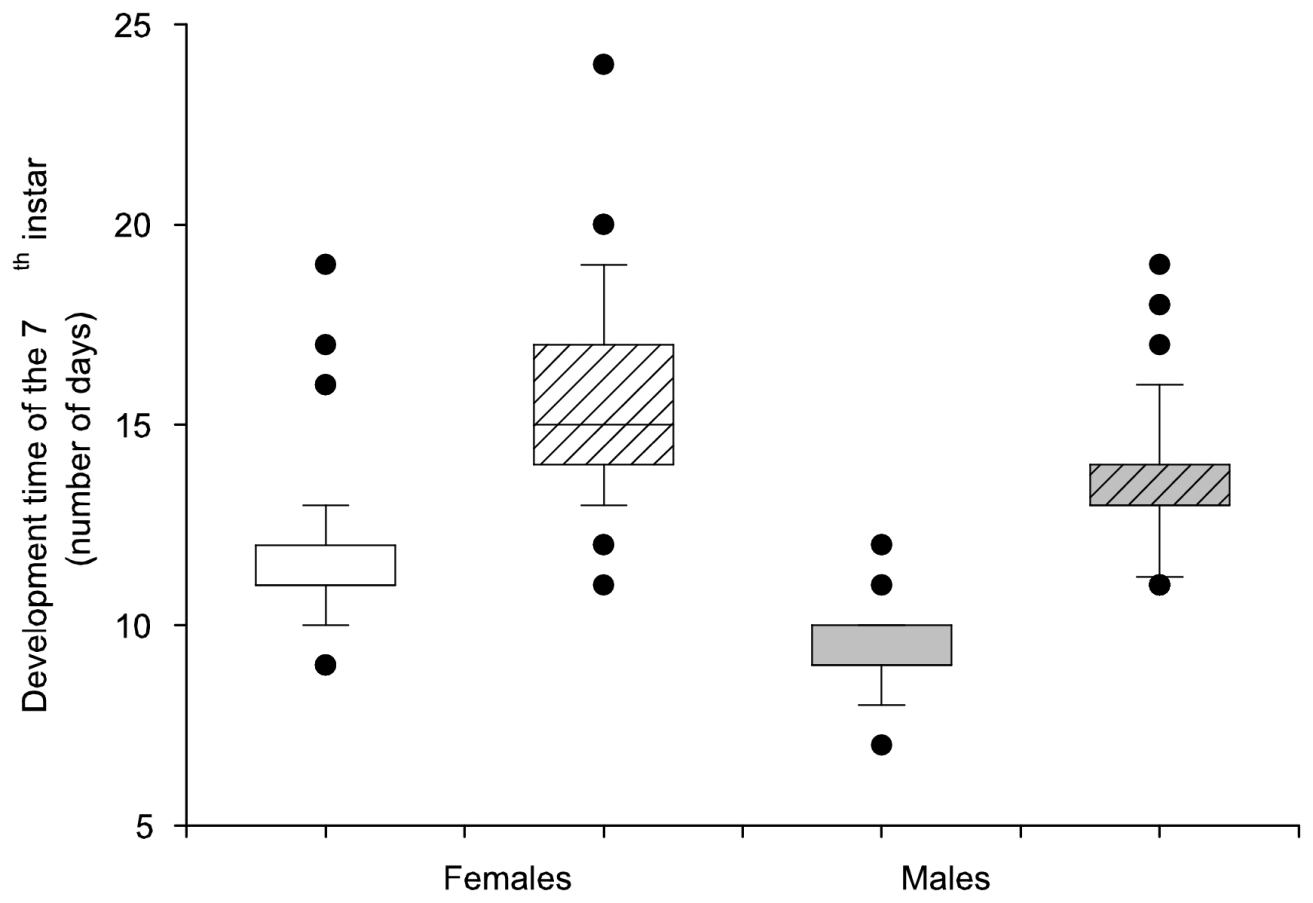
Development time (mean ± s.e.) of the final instar of larvae under control (no stripes) and food restricted (striped) feeding treatments in females (A) and in males (B).

**Figure 2:**
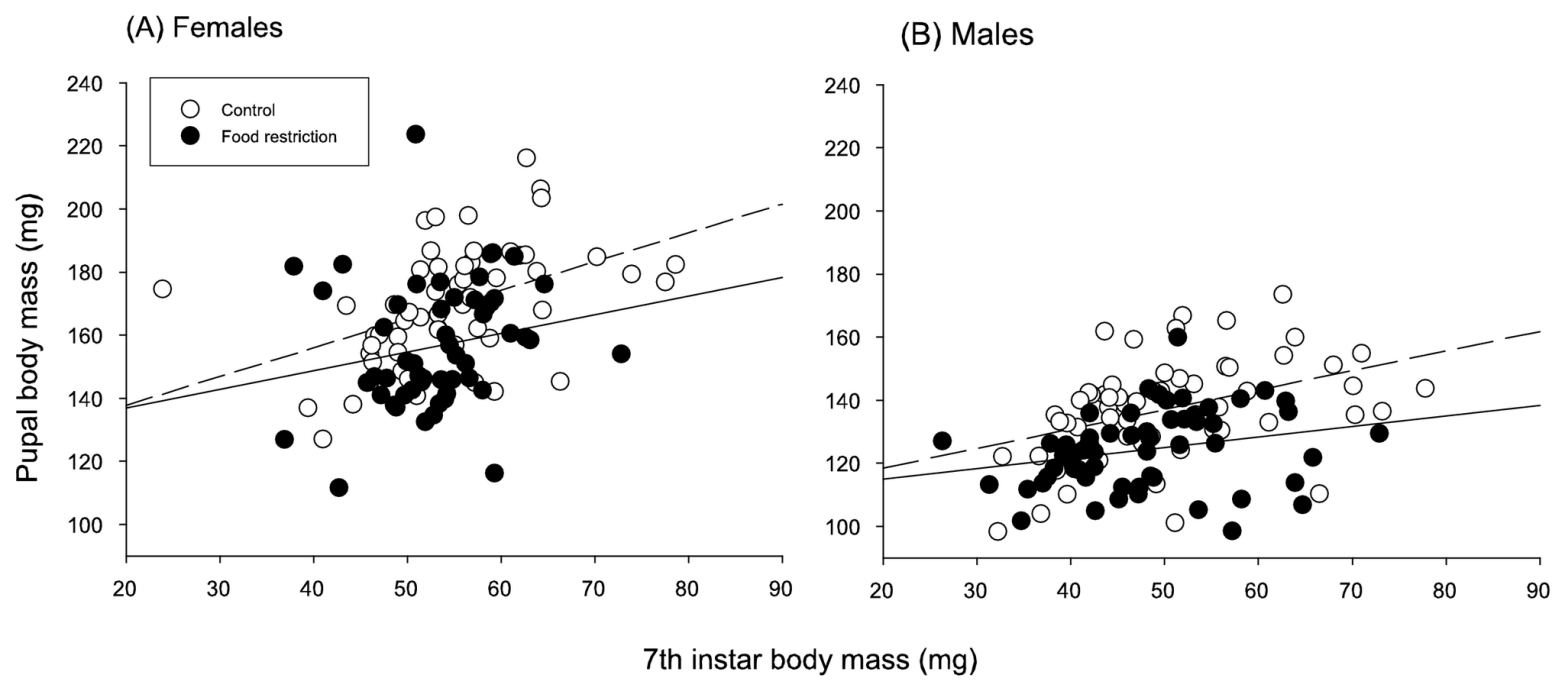
Growth in mass during the final instar of larvae under control (white circle and dashed line) and food restricted (black circle and solid line) feeding treatments in females (A) and in males (B).

The final, 7^th^ instar development time was shorter in males than in females (males: 9 ± 0.3 & 14 ± 0.3 days ± s.e. and females: 12 ± 0.3 & 16 ± 0.3 days ± s.e. for control and food restriction treatment, respectively; Table 1 & Figure 1). Males were also lighter than females as pupae (males: 137.3 ± 2.6 & 123.7 ± 2.6 mg ± s.e. and females: 170.3 ± 2.7 & 158.4 ± 2.7 mg ± s.e. for control and food restriction treatment, respectively; Table 1 & Figure 2). Experiencing three days of food restriction during the final instar prolonged the development time by four days on average (Figure 1). Individuals were not, however, able to fully compensate for the food limitation via increased development time, as they remained lighter as pupae than individuals that were fed *ad libitum* (Table 1 & Figure 2). Pupal development time was unaffected by sex and food restriction (Table 1). The effect of food restriction was not sex-specific (non-significant interaction; *P* = 0.56 *P* = 0.98, *P* = 0.68 & *P* = 0.35 for development time, weight increase in final instar, pupal mass & pupal time, respectively). 

**Table 1:** The effect of food restriction treatment and sex on larval and pupal life history traits and the effect of food restriction and encapsulation rate on adult lifespan.

	Estimate	df	*F*	*P*	Effect direction
*Larval development time*					
Sex		1, 202	96.1	< 0.0001	-*
Food restriction		1, 202	343.1	< 0.0001	+
Family	0.33 ± 19.1				
Residual	2.9 ± 0.28				
*Pupal mass*					
Sex		1, 202	270.6	< 0.0001	-*
Food restriction		1, 202	40.0	< 0.0001	-
Family	62.1 ± 27.5				
Residual	227.5 ± 22.6				
*Weight increase in final instar*					
Sex		1, 202	181.9	< 0.0001	-*
Food restriction		1, 202	27.0	< 0.0001	-
Family	32.2 ± 17.8				
Residual	236.5 ± 23.4				
*Pupal time*					
Sex		1, 202	1.6	0.213	
Food treatment		1, 202	0.03	0.861	
Family	0.01 ± 0.01				

* males had lower values

Family was included as a random factor. F statistics are given for fixed effects and Wald’s Z statistics for random effects.

### Immunity at pupal stage

**Figure 3:**
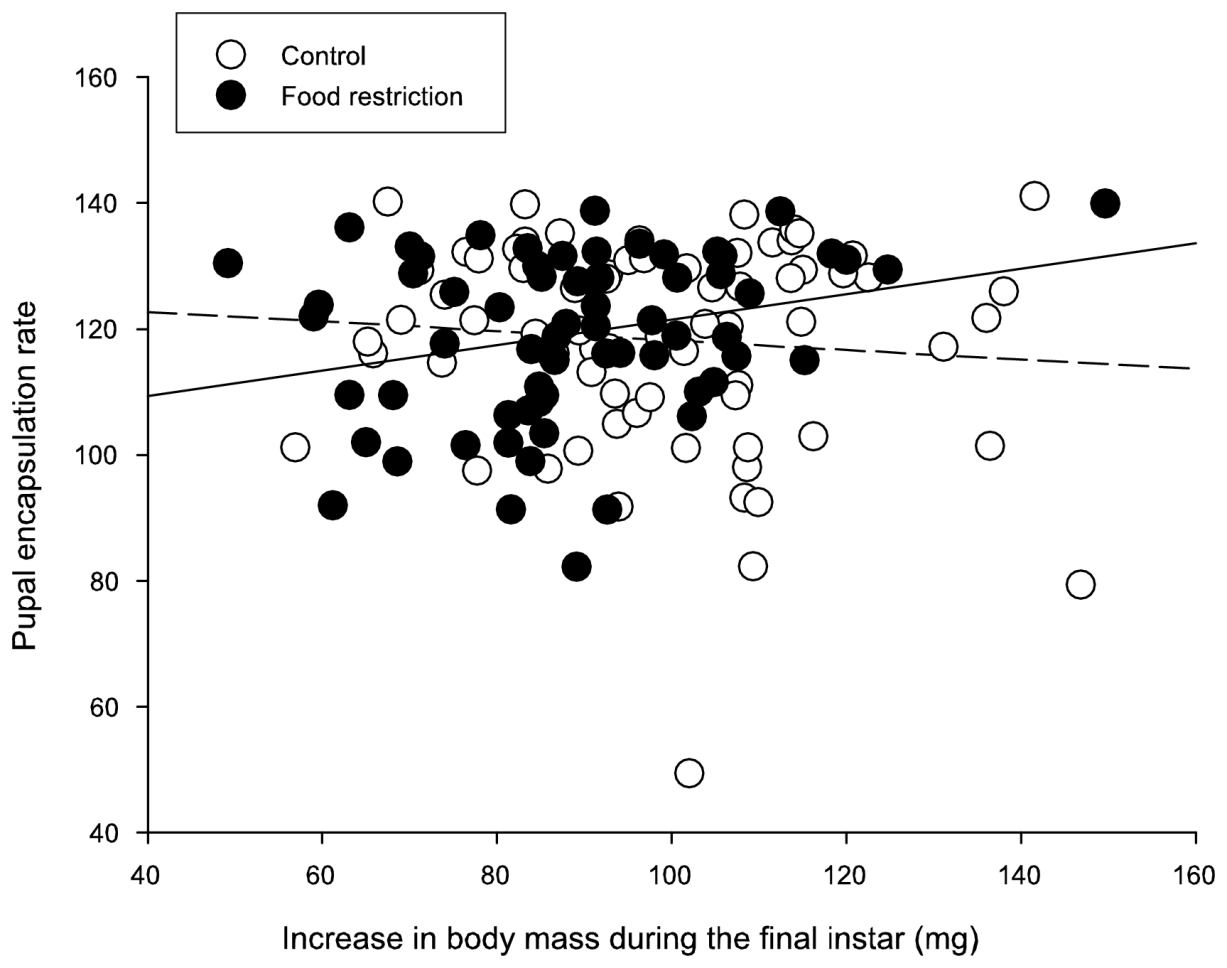
Influence of growth during the final instar on pupal encapsulation rate separately for individuals under control (white circle and dashed line) and food restricted (black circle and solid line) feeding treatments.

Encapsulation rate at pupal stage was not affected by pupal mass (*P* = 0.726). There was a trend suggesting that individuals that experienced food restriction as larvae had increased encapsulation rate (Table 2). A significant interaction between larval food treatment and increase in mass during the final instar (Table 2) indicated that individuals that grew more after the food restricted conditions had higher immunity compared with those that grew less (*P* = 0.03 for food restricted group only; Figure 3). Increase in mass during the final instar alone had no significant effect on pupal encapsulation rate (Table 2). 

**Table 2:** Factors influencing pupal and adult encapsulation rate and adult lifespan.

	Estimate	df	*F*	*P*	Effect direction
*Pupal encapsulation rate*					
Food restriction		1, 112	3.4	0.069	+
Increase in mass (7^th^ instar)		1, 112	0.8	0.397	
Food restriction x Increase in mass (7^th^ instar)		1, 112	3.9	0.050	
Family	1.0 ± 0.0				
Residual	223.7 ± 27.2				
*Adult encapsulation rate*					
Food restriction		1, 194	12.3	< 0.001	+
Forced flight		1, 194	4.3	0.040	+
Family	0.001 ± 0.0004				
Random	0.01 ± 0.001				
*Lifespan*					
Food restriction		1, 193	3.0	0.087	
Encapsulation rate		1, 193	2.2	0.139	
Food restriction x Encapsulation rate		1, 193	4.2	0.042	
Family	2.0 ± 0.0				
Residual	53.4 ± 5.1				

Family was included as a random factor. F statistics are given for fixed effects and Wald’s Z statistics for random effects.

### Metabolic rates

**Figure 4:**
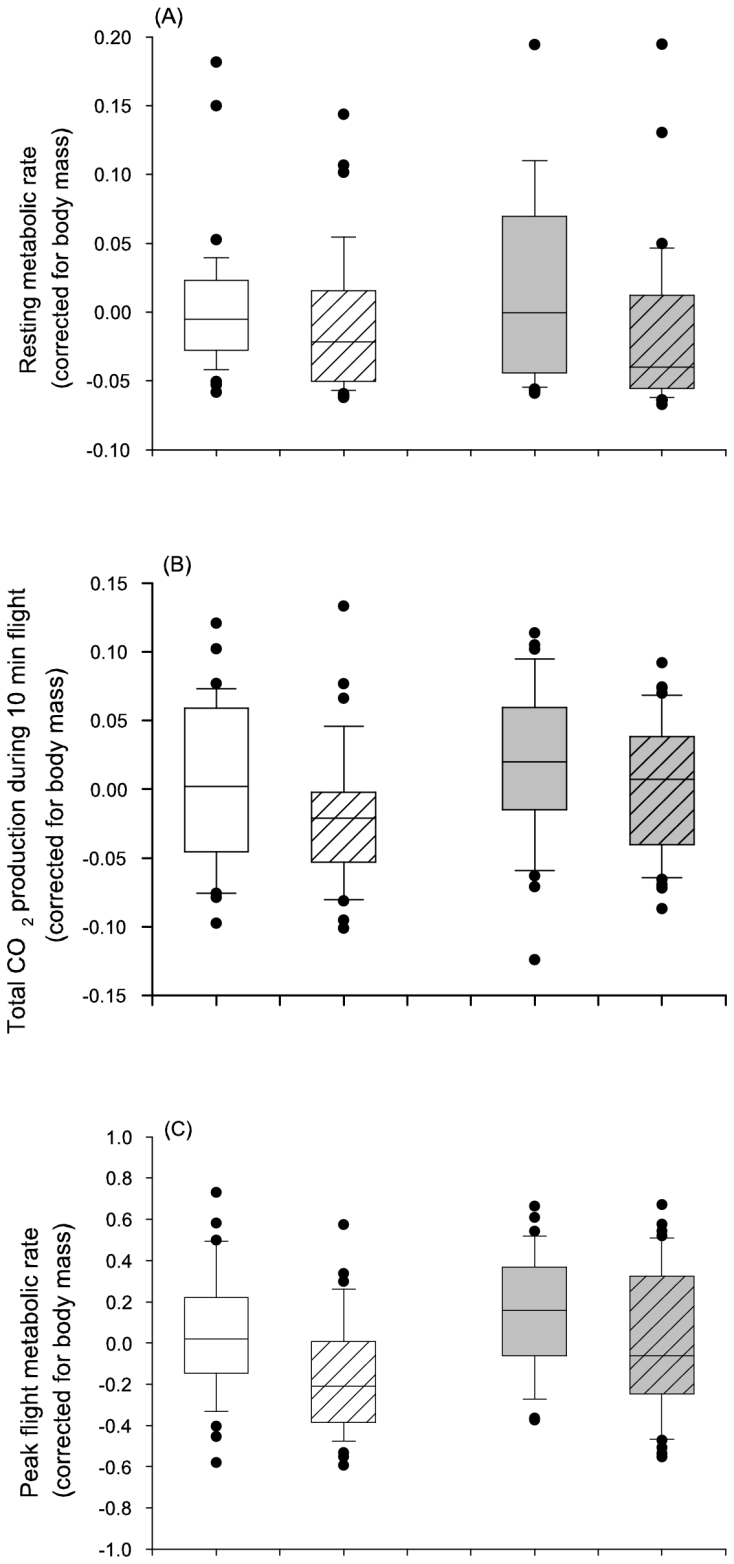
Influence (mean ± s.e.) of food conditions during development (control = no stripes, food restriction = stripes) on resting metabolic rate (RMR; A), total CO_2_ production during flight (B), and peak flight CO_2_ production separately for females (white bars) and for males (grey bars).

There was no difference in the mass-corrected resting metabolic rate (RMR) between the sexes (Table 3 & Figure 4a), whereas males had higher mass-corrected flight metabolic rate than females (both total and peak; Table 3 & Figure 4b and c). Individuals that had experienced food restriction during their development had lower RMR as well as total and peak flight metabolic rates than those fed *ad libitum* (Table 3 & Figure 4). The interaction between sex and larval treatment was not significant in any of the analyses (*P* = 0.24, *P* = 0.55 & *P* = 0.34 for RMR, flight total and peak, respectively). 

**Table 3:** The effect of food restriction and sex on metabolic rates.

	Estimate ± S.E	df	*F*	*P*	Effect direction
*RMR*					
Sex		1, 128	0.0	0.965	
Food restriction		1, 128	5.5	0.020	-
Ambient temperature		1, 128	4.4	0.04	+
Family	0.0 ± 0.0				
Residual	0.04 ± 0.005				
*Total CO_2_ produced during flight*					
Sex		1,128	5.0	0.027	+^*^
Food restriction		1,128	8.3	0.005	-
Ambient temperature		1,128	3.8	0.053	+
Family	0.0005 ± 0.0003				
Residual	0.002 ± 0.0003				
*Peak flight MR*					
Sex		1,128	5.7	0.019	+^*^
Food restriction		1,128	13.9	< 0.001	-
Ambient temperature		1,128	3.5	0.063	+
Family	0.02 ± 0.01				
Residual	0.07 ± 0.01				

* males had higher values

Family was included as a random factor. F statistics are given for fixed effects and Wald’s Z statistics for random effects. All metabolic rates are mass-corrected residuals.

### Immunity at adult stage

**Figure 5:**
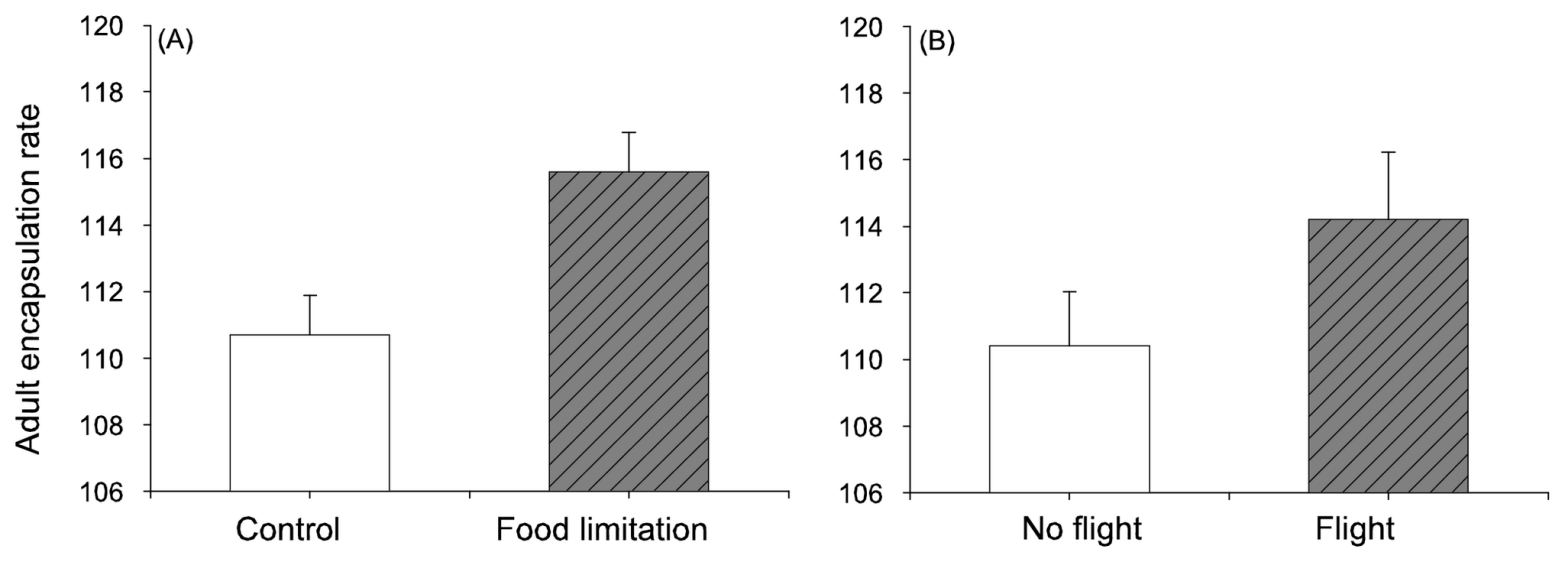
Influence of food conditions during development (A) and adult flight (B) on adult encapsulation rate (mean ± s.e.).

Individuals from the food restriction group had higher encapsulation rates at adult stage than those fed *ad libitum* (Table 2 & Figure 5a). There was no difference in encapsulation rate between the sexes (*P* = 0.27) nor did the sexes respond differently to the nutritional treatment (*P* = 0.65). There was no significant relationship between metabolic rates and encapsulation rate at adult stage (*P* = 0.50, *P* = 0.57, *P* = 0.79, respectively for RMR, total and peak flight metabolic rate). The encapsulation rate at adult stage was, however, affected by whether or not individuals were forced to fly prior the encapsulation assay. Individuals that flew had higher encapsulation rate compared with individuals that did not fly (Table 2 & Figure 5b). The interaction between food and flight treatment was non-significant (*P* = 0.28), and sex did not influence the impact of flight on immunity (*P* = 0.23). 

### Lifespan

**Figure 6:**
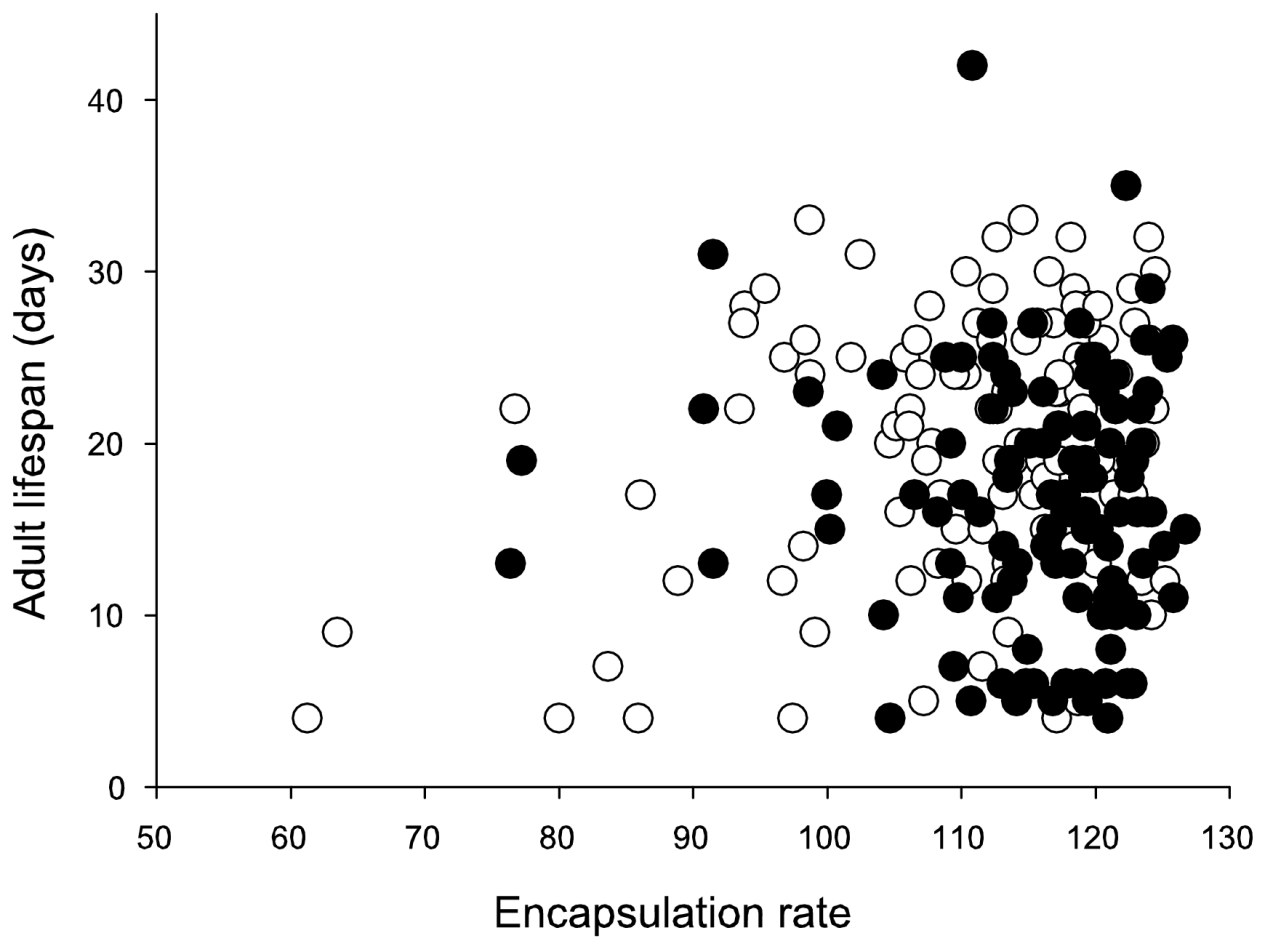
Correlations between encapsulation rate at adult stage and adult lifespan separately for individuals under control (white circle and dashed line) and food restricted (black circle and solid line) larval feeding treatments. Higher encapsulation rate correlated positively with lifespan in the control group (*r* = 0.27, *P* = 0.004), whereas in the nutritional restriction group there was no relationship between these traits (*r* = - 0.03, *P* = 0.73).

The impact of encapsulation rate on adult lifespan was dependent on the food treatment during development (Table 2). In the control group, higher encapsulation rate correlated positively with lifespan, whereas in the nutritional restriction group there was no relationship between these traits (Figure 6). Lifespan was not affected by the food treatment or encapsulation rate alone (Table 2). In addition, lifespan was not influenced by sex (*P* = 0.20) or whether or not individuals were flown (*P* = 0.34). Considering only those individuals that were assayed for flight: RMR, total and peak metabolic rate did not significantly influence lifespan (*P* = 0.79, *P* = 0.13 and *P* = 0.06), the direction of the latter two was, however, that individuals with higher flight metabolic rates lived longer.

## Discussion

Even a relatively short term food restriction during the final stages of development can have great impact on the life history of an individual, especially if individual’s resource pool and body condition are low [9,18]. In the present study, restricted feeding during development resulted in reduced pupal mass, reduced mass-corrected resting metabolic rate as well as reduced flight metabolic rate (proxy of dispersal capacity). However, no direct influence of food restriction was observed on adult lifespan and the strength of individual encapsulation rate was increased. Additionally, even though the strength of immune defence was not directly related to resting or flight metabolic rates, we found, contrary to our prediction, that flight itself induced rather than suppressed immune response in the Glanville fritillary butterfly.

Experiencing three days of food restriction, even with food available between the restricted days, had a great impact on individual’s life history in the Glanville fritillary butterfly. Development time was increased on average by four days over those individuals fed *ad libitum* throughout their development. This increased development time was not enough to fully compensate for the food restriction as individuals still remained lighter as pupae compared with individuals that developed under standard conditions. This result is interesting in the light of a previous experiment with the same species, in which otherwise identical but one day shorter food restriction resulted in similar change in development time but no difference in pupal mass [19]. There may hence be a critical threshold for food shortage after which individuals will not try to compensate for the lack of food but instead initiate pupation, even with a cost of reduced pupal mass. As in our previous study, short-term food restriction did not result in a fitness cost in terms of lifespan. It is noteworthy, however, that in the previous study food restricted individuals had reduced fecundity [19], a trait that was not assessed in the present experiment. In many organisms, including insects, restricted food intake has a positive rather than negative impact on lifespan [39]. The underlying mechanisms responsible for this are still unknown. One hypothesis is that food restriction induces responses required to survive stress, such as decreases resting metabolic rate which in turn may decrease the rate of aging [40]. In support of this pattern, we found that developmental food restriction reduced mass-corrected resting metabolic rate (RMR). However, RMR did not directly impact adult lifespan. 

Developmental food restriction had a negative impact also on the dispersal proxies, mass-corrected peak and total flight metabolic rate. This suggests that dispersal in the Glanville fritillary may be partially condition-dependent and that individuals that have experienced poor conditions during development have reduced dispersal ability. Importantly, this effect is not due to smaller body size, as the dispersal proxies were corrected for initial difference in pupal mass. Hence, food restriction is likely to influence some other aspect of individual condition (i.e. body composition) resulting in poor dispersal ability. Previous studies on the Glanville fritillary have shown dispersal tendency to be heritable [41,42] and linked with variation in a single gene, *Pgi*, encoding a glycolytic enzyme Phosphoglucose isomerase [43]. Our new results add to the growing evidence indicating the importance of both genetic [44] and environmental factors [45,46], and/or the interaction of the two [47,48] in determining dispersal ability. 

Individuals may also use poor developmental conditions (food quality or quantity and density) as cues for deteriorating environment, and alter their body composition, morphology, or behaviour in a way that enhances their ability to escape from that poor environment [49]. We found no indication of dispersal-related predictive adaptive responses in the Glanville fritillary butterfly. Such responses are likely to depend on a number of species specific factors such as life history, dispersal propensity and the spatial and temporal population structure [50], as well as the strength of resource limitation. 

We found an indication that food restricted larvae had increased encapsulation rate as pupae. This effect became more evident after the metamorphosis, when immunity was assessed at adult stage. This result is in the opposite direction to previous studies of some other insects showing that stressful conditions such as starvation reduce the strength of immune defence [51,52] but see 53. Studies in other Lepidoptera, on the other hand, have found that poor food quality [7], chemical defences in host plants [35], and heavy metal pollution [54] increase the strength of encapsulation response. It was recently also shown that in the butterfly *Bicyclus anynana* experiencing short cold thermal stress during the adult stage increased immune response (number of haemocytes) [55]. Thus, so far it seems that in Lepidoptera, stressful conditions, at least when relatively short term, in general increase immune response. Furthermore, we found that, consistent with previous studies on other taxa [16], the strength of encapsulation response was not associated with mass-corrected metabolic rate. 

As both dispersal and immune defence are known to be costly [1,11], we expected to find a trade-off between encapsulation rate and the dispersal proxies: peak flight metabolic rate (capacity) and/or total flight metabolic rate (endurance), at least for individuals that experienced food restriction during their development. In insects, intense physical activity can result in immune suppression due to competition between lipid transport and immune function for the same protein [14,56]. Similarly, in migrating birds migratory individuals have been shown to have lower innate immunity than non-migratory ones [57]. In contradiction to our general prediction, we found no relationship between peak or total flight metabolic rates and immune response in either control of food deprived butterflies. Even more surprisingly, we found that when immune response was compared between individuals that were flown and those that were not, the former had higher encapsulation rate. Based on our data it is impossible to distinguish between increased immune response after flight as a general stress response (i.e. flight itself can be viewed stressful), or as a specific response to flight and/or even dispersal. The latter could be adaptive assuming that dispersing individuals in the wild may be faced with higher pathogen and/or parasite risk during the dispersal process itself or in the new habitat that they disperse to. Increased infection risk during dispersal or settlement could result from individuals being generally stressed or weak due to high energetic costs of dispersal. Alternatively, increased susceptibility to infection may arise from the fact that dispersers are exposed to a higher number of new habitats and maybe to new genotypes of pathogens to which they are not locally adapted. There is some evidence that dispersal interacts with the strength of immune defence. For example, using the damselfly *Calopteryx virgo*, it was found that activation of the immune system increased dispersal tendency [12]. Consistently, in the Monarch butterfly, *Danaus plexippus*, genes related to innate immunity are up-regulated in non-reproductive (juvenile-hormone deficient) migrants [58]. Importantly, the relationships between dispersal, immunity and condition may also vary across populations, as was shown in the mormon cricket, for which the influence of nutrition on immunity differed between two migratory populations [6]. Immune responses, in general, are likely to vary across local populations due to coevolutionary interactions such as differences in pathogen and parasitoid prevalence [59]. 

It is noteworthy that the immune response to flight was assessed almost immediately after flight and hence it is possible that the observed increased immune response is not long lasting. This idea is supported by the result that increased immune response, which presumably is energetically costly, did not have a negative effect on lifespan. Finally, we did not find a negative correlation between immune defence and flight metabolic rates, even on individuals with fewer resources (i.e. food deprived during development). It would be interesting in the future to assess the impact of adult nutrition [60], as the trade-offs may be evident only under unfavourable adult conditions [2] but see 61. 

The effects of nutrition restriction and flight that we observed may be specific to the immune defence measured, and hence somewhat different results may have been obtained if we would have measured other responses than encapsulation. For example, it was recently shown in mice that selection for maximal metabolic rate supresses innate (cytokine production) but not adaptive immune function (antibody production) [62]. Similarly, in the present study immune response at adult stage was assessed on young and non-reproductive individuals, and hence future studies should also assess the possible trade-offs in older individuals at different reproductive stages when energy demands may be higher and trade-offs more evident. Finally, as our study was conducted in the laboratory, individuals were not exposed to the normal range of pathogens. The influence of nutritional restriction under more variable pathogen conditions could be very different [28]. 

In summary, we have shown that environmental conditions during development can have a great impact on adult life history, namely on metabolic rates, including a proxy for dispersal propensity, and encapsulation rate in the Glanville fritillary butterfly. Stressful events during both development (food restriction) and adult life (forced flight) induce an increased immune response, possibly allowing individuals to cope with later stressful events. Identifying the actual mechanisms that link both developmental conditions and flight to encapsulation rate, our measure of immune response, will remain a task for the future.
